# How Do Structurally Distinct Compounds Exert Functionally Identical Effects in Combating Obesity?

**DOI:** 10.3389/fphar.2018.00069

**Published:** 2018-02-07

**Authors:** Zhi-Jun Deng, Ruo-Xuan Liu, A-Rong Li, Jie-Wen Guo, Qing-Ping Zeng

**Affiliations:** ^1^Guangzhou Hospital of Traditional Chinese Medicine, Guangzhou, China; ^2^Tropical Medicine Institute, Guangzhou University of Chinese Medicine, Guangzhou, China

**Keywords:** obesity, inflammation, weight reduction, mitochondria, nitric oxide

## Abstract

Although the concept of inflammatory obesity remains to be widely accepted, a plethora of antibiotics, anti-inflammatory agents, mitochondrial uncouplers, and other structurally distinct compounds with unknown mechanisms have been demonstrated to exert functionally identical effects on weight reduction. Here we summarize a universal mechanism in which weight loss is modulated by mitochondrial biogenesis, which is correlated with conversion from the mitochondria-insufficient white adipose tissue to the mitochondria-abundant brown adipose tissue. This mechanistic description of inflammatory obesity may prove useful in the future for guiding pathology-based drug discovery for weight reduction.

## Introduction

Obesity is closely associated with a series of health conditions, including type 2 diabetes mellitus (T2DM), cardiovascular disease, and even cancers that confer a higher risk of mortality. The current prevalence and trend data indicate that about 2 billion people globally are overweight and one third of these obese, and these conditions are linked to a variety of physical and psychosocial health concerns (Seidell and Halberstadt, 2015). Although it remains controversial, the American Medical Association (AMA) has declared obesity to be a disease (Beal, 2013). Some specialists have argued that obesity is not a disease, but a risk factor for other chronic diseases (Katz, 2014). Primary care physicians have also been reported to hold divergent opinions on whether obesity should be considered a risk factor or a disease (Funk et al., 2016). Clinically, some obese people without inflammation are sensitive to insulin and do not develop T2DM, whereas others with inflammation are resistant to insulin and develop T2DM. Therefore, a distinct classification into non-inflammatory/healthy obesity and inflammatory/unhealthy obesity has been independently suggested by multiple authors (Hawkesworth et al., 2013; Jais et al., 2014).

Although the concept of inflammatory obesity has yet to be commonly accepted, it has been highlighted with respect to possible mechanisms that cryopyrin/NLRP3 inflammasome-induced adipose macrophages in mice facilitate weight gain by upregulating monoamine oxidase A (MAOA), which degrades the catecholamine noradrenaline and hence decreases the levels of the critical lipolytic enzymes adipose triglyceride lipase (ATGL) and hormone-sensitive lipase (HSL) (Camell et al., 2017).

In support of the inflammatory origin of obesity, both antibiotics and anti-inflammatory agents have been documented to exert weight-reducing effects by eradicating pathogenic infection and abrogating pro-inflammatory responses, respectively, as described in detail below.

## Weight Reduction Using Antibiotics

The recent finding that the overgrowth and excessive metabolic activity of gut microbiota flora raise the risk of developing obesity and related metabolic disorders suggests that manipulating the dysbiotic gut microbiota population using various antimicrobial strategies may be beneficial for the management of obesity and the prevention of obesity-associated malfunctional conditions (Murphy et al., 2013). Similarly, Angelakis et al. (2013) have reviewed the effects of antibiotics on the gut microbiota of humans and animals and discussed the potential therapeutic use of antibiotics for weight loss in the former.

For example, it has been revealed that the conversion of white adipose tissue (WAT) to brown adipose tissue (BAT) is promoted by antibiotic treatment or in germ-free mice (Suárez-Zamorano et al., 2015). Consequently, glucose tolerance and insulin sensitivity are improved in obese leptin-deficient (*ob*/*ob*) mice and mice fed with a high-fat diet (HFD). Conversely, these indices are reversed by recolonization of the antibiotic-treated or germ-free mice with microbes.

In another investigation, it was demonstrated that *Methanobrevibacter smithii* reduction and breath methane eradication by a 10-day antibiotic therapy regimen significantly decreases the serum levels of total cholesterol and low-density lipoprotein in prediabetic subjects with obesity, as well as leading to lower insulin and glucose levels (Mathur et al., 2016).

## Weight Reduction Using Anti-Inflammatory Agents

Salicylate, an active metabolite of the non-steroidal anti-inflammatory drug (NSAID) aspirin, can reverse HFD-induced obesity and insulin resistance by the inhibition of nuclear factor kappa B (NF-κB) (Yuan et al., 2001). Exposure to the anti-inflammatory agents aspirin or statins is associated with weight loss in T2DM patients (Boaz et al., 2009). The immunosuppressant rapamycin also exhibits a weight-reducing effect, normalizing serum leptin and alleviating obesity in aged rats (Scarpace et al., 2016), perhaps by inhibiting NF-κB activation and repressing pro-inflammatory signaling (Chen et al., 2014).

Surprisingly, the antimalarial drug artemisinin and the antidiabetic drug metformin have been demonstrated to display an unexpected weight-reducing effect (see below). Interestingly, artemisinin was previously found to inhibit NF-κB signaling (Aldieri et al., 2003). Similarly, metformin was also recently shown to suppress signal transducer and activator of transcription (STAT) signaling in addition to NF-κB signaling (Saengboonmee et al., 2017).

In the obese mouse model described above (Camell et al., 2017), the deletion of cryopyrin/NLRP3 in the inflammation-induced adipose macrophages was found to restore noradrenaline-induced lipolysis by downregulating growth differentiation factor-3 (GDF3). Furthermore, the deletion of GDF3 in the inflammasome-activated macrophages improved lipolysis by decreasing MAOA levels. The inhibition of MAOA reverses noradrenaline reduction and restores lipolysis, leading to increased ATGL and HSL levels in adipose tissue.

## Weight Reduction Using Mitochondrial Uncouplers

Although the increased lipid degradation upon inflammasome inhibition can be reasonably explained as described above, the mechanism of how fatty acids be degraded completely remains unknown. Nevertheless, it can be deduced that the mitochondrial dynamics from a greater number of mitochondria in BAT to fewer mitochondria in WAT should affect the body weight, i.e., obese or lean, because the sequential β-oxidation of fatty acids principally occurs within the mitochondria.

As a mitochondrial uncoupler that disassociates electron transport from oxidative phosphorylation through leakage of protons across the inner mitochondrial membrane, 2,4-dinitrophenol (DNP) has been widely used as an effective weight loss drug since the early 1930s (Goldgof et al., 2014). The controlled release of DNP not only reduces hypertriglyceridemia, insulin resistance, hepatic steatosis, and diabetes in the HFD-induced obesity rat model, but also normalizes plasma transaminase levels, ameliorates liver fibrosis, and improves hepatic protein synthesis function in a methionine/choline-deficient rat model of non-alcoholic steatohepatitis (Gao et al., 2015; Perry et al., 2015).

## Weight Reduction Using Other Weight-Reducing Drugs With Unknown Mechanisms

Apart from antibiotics, anti-inflammatory agents, and mitochondrial uncouplers, a plethora of other structurally unrelated compounds with unknown mechanisms of action, such as the dimethylbiguanide metformin, the trihydroxystilbene resveratrol, and the sesquiterpene artemisinin, also exert some degree of weight-reducing effects.

In one study, the mean weight loss in the metformin-treated group over 6 months was found to be 5.8 ± 7.0 kg, whereas untreated controls (non-diabetic individuals with obesity) gained 0.8 ± 3.5 kg on average (Seifarth et al., 2013). In particular, patients with severe insulin resistance lost significantly more weight compared with insulin-sensitive patients.

In rats fed a resveratrol-containing diet, it was found that abdominal adipose accumulation was markedly prevented, fat metabolism and sparing actions for carbohydrates and proteins were partially enhanced, and adipose carnitine palmitoyltransferase mRNA levels were significantly elevated (Nagao et al., 2013).

C3H10T1/2 cells treated with artemether, an artemisinin derivative, were found to display a typical thermogenic morphology: smaller adipocytes with plurilocular lipid droplets (Lu et al., 2016). Mitochondrial biogenesis-related genes such as *UCP1*, *PGC-1α*, *PRDM16*, and *CytC* were also upregulated by artemether in a dose-dependent manner.

## Suggested Mechanism Underlying Mitochondria-Dependent Adipogenesis and Adipolysis

To account for weight gain and weight loss in a mitochondria-dependent manner, we suggest here a mechanism that we refer to as “high/low-level nitric oxide (NO)-switched adipogenesis/adipolysis,” in which a pro-inflammatory signal upregulates *NOS2*/inducible nitric oxide synthase (iNOS) to trigger a potent NO burst, block mitochondrial respiration, and facilitate lipogenesis, whereas an anti-inflammatory response upregulates *NOS3*/endothelial nitric oxide synthase (eNOS) to maintain mild NO release, prompt mitochondrial biogenesis, and enhance lipolysis.

In terms of the evidence supporting such a mechanism, it has been established that high levels of NO inhibit cell respiration, whereas slow and small-scale NO release stimulates mitochondrial biogenesis by binding to cytochrome *c* oxidase (COX) in mitochondria (Nisoli and Carruba, 2006). For example, calorie restriction enhances mitochondrial biogenesis, which is initiated by eNOS-derived low-level NO (Nisoli et al., 2005). However, it remains unknown whether metformin, resveratrol, or artemisinin can also generate NO, interrupt electron transport, trigger mitochondrial biogenesis, or accelerate energy expenditure.

## Emerging Evidence Supporting the Putative Weight-Reducing Mechanism

Through mitochondrial uncoupling, DNP leads to the synchronous increases of adenosine monophosphate (AMP) and oxidized nicotinamide adenine dinucleotide (NAD^+^) levels (Korde et al., 2005). Increases in AMP and NAD^+^ levels can activate AMP-activated kinase (AMPK) and NAD^+^-dependent deacetylase sirtuin-1 (SIRT1), respectively, which in turn activate peroxisome proliferator-activated receptor-γ co-activator 1α (PGC-1α) for mitochondrial biogenesis (Rodgers et al., 2005; Lee et al., 2006).

Although metformin was previously identified as a classic activator of AMPK (Martin-Montalvo et al., 2013), it can also target mitochondria to inhibit NADH dehydrogenase and adenosine triphosphate (ATP) synthase, thereby triggering mitochondrial biogenesis (Bridges et al., 2014). Additionally, the inhibition of the mitochondrial respiratory function by metformin results in the upregulation of fibroblast growth factor 21 (FGF21), which also possesses anti-obesity and anti-diabetes effects (Kim et al., 2013).

Resveratrol is conventionally classified as an activator of SIRT1 (Blagosklonny, 2010), but it can also induce mitochondrial biogenesis in a manner dependent upon NO production, cyclic guanosine monophosphate (cGMP) biosynthesis, heme oxygenase 1 (HO-1) activation, and carbon monoxide (CO) generation (Kim et al., 2014). Actually, resveratrol has been previously reported to induce HO-1 expression via nuclear erythroid 2-related factor 2 (Nrf2)/antioxidant response element (ARE) activation in neuronal PC12 cells (Chen et al., 2005). HO-1 induction can reduce the production of proinflammatory cytokines such as tumor necrosis factor alpha (TNF-α) and interleukin 6 (IL-6) from adipocytes and macrophages by inhibiting the activation of inflammatory signaling molecules including NF-κB and Janus kinase (JNK) (Tu et al., 2014). However, the exact mitochondrial target of resveratrol has yet to be identified.

By targeting cytochrome c1 and NADH dehydrogenase [ubiquinone] flavoprotein 1, artemisinin resembles the NO donor nitroglycerin and exerts anti-inflammatory effects, downregulates *NOS2*/iNOS expression, maintains stable NO release, and augments adipose mitochondrial functions that necessitate adipolysis for weight loss (Gao et al., unpublished). Interestingly, it was found that NO generation is well-correlated with ATP production, *NOS3*/eNOS upregulation, and mitochondrial biomarker overexpression upon treatment with artemisinin or nitroglycerin, suggesting that artemisinin- or nitroglycerin- generated NO prompts adipose degradation and energy expenditure by triggering mitochondrial biogenesis.

It has recently been reported that AMPK catalyzes the phosphorylation of JAK and inhibits JAK/STAT signaling (Rutherford et al., 2016), which implies that mitochondrial uncouplers also resemble antibiotics or anti-inflammatory agents to exert anti-inflammatory effects by activating AMPK. Conversely, it was also shown that AMPK activates eNOS via the AMPK→Rac1→Akt→ eNOS pathway (Levene et al., 2007), thereby indicating that antibiotics and anti-inflammatory agents also result in mitochondrial uncoupling, by which they influence mitochondria through the inhibition of iNOS-generated high-level NO and the activation of eNOS-synthesized low-level NO.

Accordingly, mitochondrial dysfunction may lead to inflammation because a mitophagy/autophagy blockade causes the accumulation of damaged and reactive oxygen species (ROS)-generating mitochondria, which in turn activates the NLRP3 inflammasome to sense mitochondrial dysfunction. This pathway would explain the possible association of mitochondrial damage with inflammatory diseases (Zhou et al., 2011).

**FIGURE 1:**
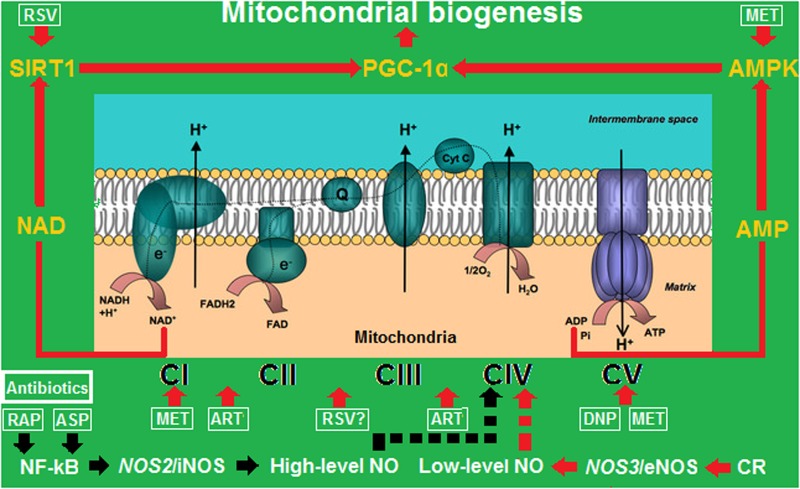
Mitochondrial biogenesis driven by calorie restriction (CR), anti- inflammatory agents, and energy uncouplers by turning off/on the iNOS/eNOS switch. ART, artemisinin; ASP, aspirin; DNP, 2,4-dinitrophenol; MET, metformin; RAP, rapamycin; RSV, resveratrol. CI–CV represent mitochondrial complexes I–V. The red arrows represent activation or upregulation, and the black arrows represent inactivation or downregulation. While low-level NO transiently binds to CIV to trigger mitochondrial biogenesis, high-level NO permanently binds to CIV to block mitochondrial respiration.

In summary, the roles of antibiotics, anti-inflammatory agents, and mitochondrial uncouplers in combating obesity are linked by the common effects of increasing eNOS-derived low-level NO to enter mitochondria, target mitochondrial complexes, and interfere with mitochondrial functions, which results in enhanced mitochondrial biogenesis, increased fatty acid oxidation, and accelerated weight reduction (**Figure 1**).

The above mechanistic definition of the concept of inflammatory obesity is anticipated to assist in the discovery of pathology-based weight-reducing drugs, which will be beneficial for combating obesity and therefore mitigating the effects of obesity-related metabolic disorders in the near future.

## Author Contributions

J-WG and Q-PZ wrote the manuscript. All authors reviewed and approved the final version of the manuscript.

## Conflict of Interest Statement

The authors declare that the research was conducted in the absence of any commercial or financial relationships that could be construed as a potential conflict of interest.
